# Biosynthesis Pathways, Transport Mechanisms and Biotechnological Applications of Fungal Siderophores

**DOI:** 10.3390/jof8010021

**Published:** 2021-12-28

**Authors:** Lorenzo Pecoraro, Xiao Wang, Dawood Shah, Xiaoxuan Song, Vishal Kumar, Abdul Shakoor, Keshawanand Tripathi, Pramod W. Ramteke, Rupa Rani

**Affiliations:** 1 School of Pharmaceutical Science and Technology, Tianjin University, 92 Weijin Road, Tianjin 300072, China; wang_xiao1996@163.com (X.W.); dawoodshah616@gmail.com (D.S.); harusong@163.com (X.S.); abdul_shakoor954@yahoo.com (A.S.); ruparani719@gmail.com (R.R.); 2 Institute of Biotechnology and Genetic Engineering, The University of Agriculture Peshawar, Peshawar 25000, Pakistan; 3 Department of Food Science and Technology, Yeungnam University, Gyongsan 38541, Korea; vkaggarwal180@gmail.com; 4 College of Geography and Environmental Science, Henan University, Kaifeng 475004, China; 5 Center for Conservation and Utilization of Blue-Green Algae, ICAR-Indian Agricultural Research Institute, New Delhi 110012, India; tripathikn009@gmail.com; 6 Faculty of Life Sciences, Mandsaur University, Mandsaur 458001, India; pwramteke@gmail.com; 7 Department of Environmental Science and Engineering, Indian Institute of Technology (Indian School of Mines), Dhanbad 826004, India

**Keywords:** fungal cytoplasm proteins, iron uptake, iron-binding molecules, siderophore biosynthesis, hydroxamate, siderophore-Fe chelators mannoproteins, biotechnology, medicine, biocontrol, bioremediation

## Abstract

Iron (Fe) is the fourth most abundant element on earth and represents an essential nutrient for life. As a fundamental mineral element for cell growth and development, iron is available for uptake as ferric ions, which are usually oxidized into complex oxyhydroxide polymers, insoluble under aerobic conditions. In these conditions, the bioavailability of iron is dramatically reduced. As a result, microorganisms face problems of iron acquisition, especially under low concentrations of this element. However, some microbes have evolved mechanisms for obtaining ferric irons from the extracellular medium or environment by forming small molecules often regarded as siderophores. Siderophores are high affinity iron-binding molecules produced by a repertoire of proteins found in the cytoplasm of cyanobacteria, bacteria, fungi, and plants. Common groups of siderophores include hydroxamates, catecholates, carboxylates, and hydroximates. The hydroxamate siderophores are commonly synthesized by fungi. L-ornithine is a biosynthetic precursor of siderophores, which is synthesized from multimodular large enzyme complexes through non-ribosomal peptide synthetases (NRPSs), while siderophore-Fe chelators cell wall mannoproteins (FIT1, FIT2, and FIT3) help the retention of siderophores. *S. cerevisiae*, for example, can express these proteins in two genetically separate systems (reductive and nonreductive) in the plasma membrane. These proteins can convert Fe (III) into Fe (II) by a ferrous-specific metalloreductase enzyme complex and flavin reductases (FREs). However, regulation of the siderophore through Fur Box protein on the DNA promoter region and its activation or repression depend primarily on the Fe availability in the external medium. Siderophores are essential due to their wide range of applications in biotechnology, medicine, bioremediation of heavy metal polluted environments, biocontrol of plant pathogens, and plant growth enhancement.

## 1. Introduction

Iron plays a vital role in the growth and development of living organisms, and it is one of the most abundant elements found on earth [1]. Mineral bioweathering is important in soil ecosystems because it increases the availability of iron for colonizing organisms, which is limited in oxygenic environments [2,3]. Indeed, Fe is converted into insoluble oxyhydroxide polymers under aerobic conditions at biological pH (oxidized form) [4]. Plants require (10^−5^–10^−7^ M) of Fe (II) for growth and development, whereas the solubility of Fe (III) in nature is 10^–17^ M at pH 7 [5]. During the evolution of life on earth, the majority of iron was in insoluble Fe (III) forms. Therefore, microorganisms including fungi, bacteria, and cyanobacteria adapted to low iron availability levels and overcame iron deficiency by synthesizing siderophores [6,7,8]. Siderophores are low molecular weight compounds (200–2000 Da) produced by different microbes, which chelate the iron from different habitats [9]. Scientific data have shown the ubiquitous presence of siderophores in plants and microbes. A significant number, i.e., 500 fungal and bacterial siderophores, are documented [10]. Furthermore, mammalian siderophores have also been reported [11]. Several laboratory methods, including spectrophotometric titration, electrophoretic mobility, mass spectrometry, acid hydrolysis, and biological activity tests are used to characterize siderophores [12]. 

Fe plays an essential role in vital functions, including photosynthesis, respiration, synthesis of DNA, RNA, proteins, and enzyme cofactors [13]. In human serum, transferrin is an iron transport protein that maintains Fe (III) concentration (10–24 M) and blocks the entry of pathogens [14]. Fungi and other microorganisms adopt various strategies for iron acquisition from the extracellular environment by (i) use of metal ion transporters [15], (ii) acquisition from heme and heme containing-proteins [16], (iii) acquisition from transferrin, lactoferrin, and ferritin [17,18], (iv) use of reductive systems of iron uptake, and (v) siderophore-mediated iron transport [8]. Siderophore-mediated Fe-scavenging is an essential process in soil ecosystems that improves the bioavailability of iron derived from mineral dissolution via bioweathering. Siderophores primarily scavenge iron through complex formation with other metals such as molybdenum and cobalt [19]. These compounds promote plant growth and play an important role in pathogen biocontrol [20] and bioremediation of metal-polluted environments [21].

Fungal siderophores are very diverse and show striking structures. Fungi usually produce hydroxamate and carboxylate siderophore types, which have been primarily studied in *Aspergillus* species. For instance, *A. fumigatus* and *A. nidulans* synthesize about 55 types of siderophores. Both species live as saprotrophs, contributing to maintaining carbon and nitrogen cycles in the environment [8]. Many fungi can produce more than one siderophore type, especially under low iron availability. *Aspergillus fumigatus* often produces a hydroxamate siderophore and triacetyl fusarine C (TAFC) for tapping extracellular iron [22]. This fungus can also secret a siderophore called ferricrocin for mobilization and distribution of hyphal iron, and intracellular iron storage. Besides, *A. fumigatus* synthesizes the conidial siderophore ‘hydroxyferricrocin’ for storing iron in its conidia to support the germination process and reduce oxidative stress [23]. *Aspergillus nidulans* has been reported to produce two main siderophores, ferricrocin (Frr) and ferrihordin. A twenty-four hour culture of *A. nidulans* produced an unacetylated form of TAFC, which is known as fusigen. In contrast, an older strain (48 h) yielded acetylated TAFC due to breakdown and uptake of fusigen [24]. Another fungus, *Wolfiporia cocos*, known as a brown-rot fungus, may produce different types of catecholate siderophores [25]. According to Haselwandter et al. [26], the two ubiquitous ectomycorrhizal basidiomycetes *Laccaria laccata* and *L. bicolor* can produce linear ester-containing Fsg siderophores, i.e., CPG, Frr and TAFC, apart from the hydroxamates.

Many researchers are now interested in knowing how some fungal strains have evolved to produce different types of siderophores, what are the suitable conditions for the production of siderophores, how siderophores, in general, can contribute to fungal survival, and what are the structural differences between fungal siderophores. Indeed, many studies have used structural and stereochemical analyses to describe the properties of fungal siderophores, while attempts have been made to understand the production, recognition, and transportation mechanism of siderophores in different fungal species. Some members of siderophore classes have been characterized by crystalline structures, the absolute metal center configuration, and conformation in solutions [8,27]. Understanding of chemical and structural properties and transport mechanisms of fungal siderophores has also inspired many researchers. Ferrichromes (FRC), for example, are the best studied siderophores and are now regarded as biomolecules. However, there is still a dearth of knowledge on how siderophores are designed for transport. Except in plants, nothing is known about the possible membrane-located transport system for siderophores in the fungal plasma membrane.

Further studies are still needed to describe in detail, or confirm, the protein nature of many fungal siderophores. However, some studies have already clarified the energy requirement for siderophore-mediated iron transport in fungi. There is evidence suggesting that the transportation of siderophores can be mechanically transferred across the cytoplasmic membrane. However, it is still not fully known yet to what extent this is possible.

## 2. Overview of Fungal Siderophores

**Figure 1:**
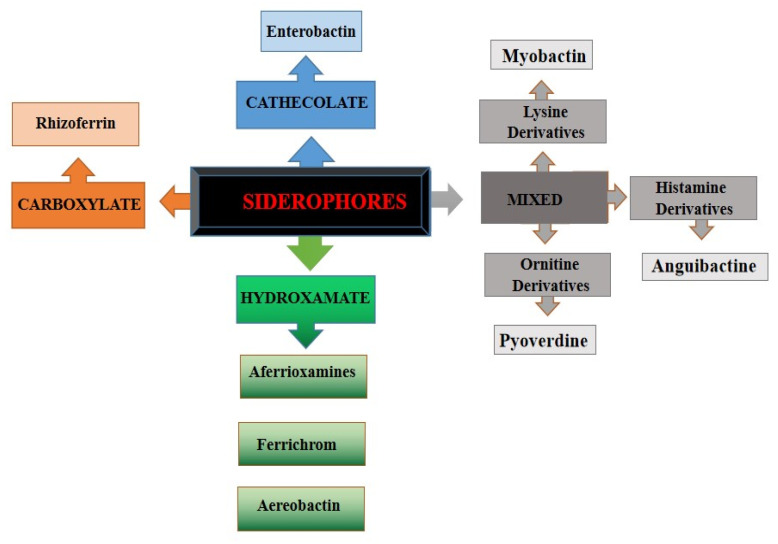
General groups of siderophores.

Fungi are heterotrophic eukaryotic organisms [28]. Among them, saprobic fungal species are decomposers that break down and feed on decaying organic matter. Many fungi show two major responses to iron concentration in the environment: siderophore synthesis under iron stress, and a high-affinity ferric iron reductase [29]. On the basis of chemical interaction sites, siderophores have been classified into two major groups, i.e., Enterobactin and Hydroxamates. Enterobactin is regarded as a good iron chelator, which has the ability to interact with iron and catecholate hydroxyl groups. Hydroxamates are unique, due to the presence of N-hydroxylated amide bonds. The hydroxamate siderophores, such as FRC, are commonly produced by fungi. Siderophores that are synthesized by microbes and plants are classified based on coordinating groups and Fe binding system, including (i) phenolates, (ii) hydroxamates, (iii) polycarboxylates (Table 1). Common types of siderophores are hydroxamates, catecholates, and carboxylates [30]. Nitrogen, oxygen, and sulfur atoms can take part in iron coordination in the carboxylate group of siderophores [31,32]. Another group of siderophores is known as the mixed type. Mixed type siderophores do not belong to hydroxamate and the aromatic hydroxyl category. These hybrid types of siderophores are classified based on the position of the Fe (III) binding group. Mixed-type siderophores can bind salicylic acid and nitrogen [4]. Common examples of mixed type siderophores are (i) lysine derivatives, such as myobactin, (ii) ornithine derivatives, including pyoverdine, and (iii) histamine derivatives, such as anguibactin. Other examples are thiazoline, oxazoline, and pyoverdine. Pyoverdine, in particular, has been reported as a signaling molecule in the bacterium *Pseudomonas aeruginosa* [33] and as an inhibitor molecule against zinc-containing matrix metalloproteinases (MMPs), which often degrade in extracellular matrixes [34]. The main groups of siderophores are itemized in Figure 1. Many phytopathogenic fungi synthesize unique compounds to chelate iron, but also produce phytotoxins. Fungi mainly synthesize hydroxamate-type siderophores (derived from the nonproteinogenic ornithine amino acid) (Table 1).

**Figure 2:**
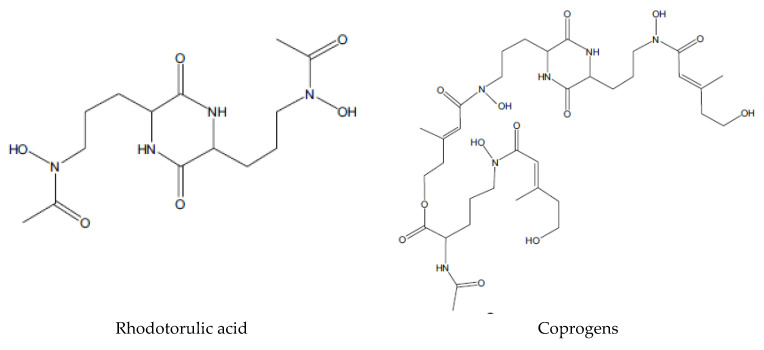

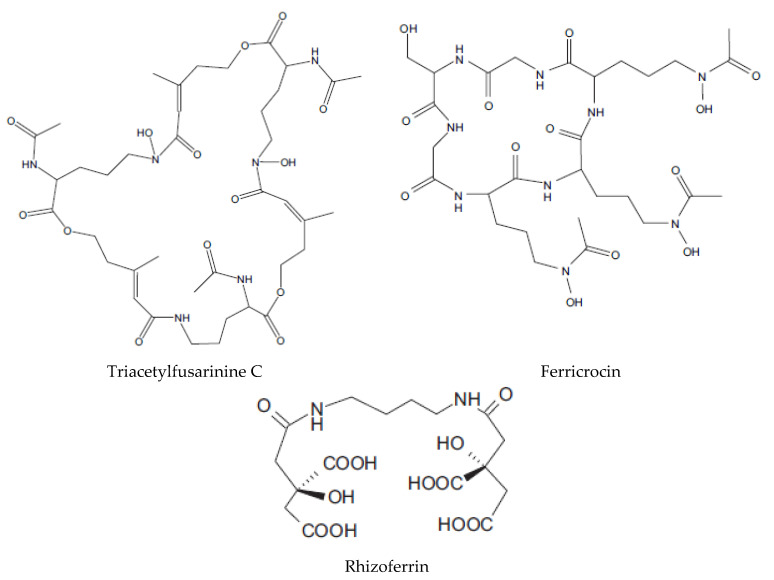
Structure of fungal siderophores.

Hydroxamates are further categorized into three groups: (i) fusarinines (FSR), (ii) coprogens (CPG), (iii) FRC, with a few exceptions, such as the rhizoferrin (Figure 2). Rhizoferrin is a carboxylate-type siderophore produced by certain zygomycetes. Siderophore synthesis by fungi depends on the nutrient medium and culture conditions. Hydrophilic siderophores are derived from the common structural unit N^δ^-hydroxyornithine. In fungi, they consist of hydroxylated and alkylated ornithine amino acid, while in bacteria, they are acylated and hydroxylated alkylamines [37], represented by N^6^-acyl-N^6^-Hydroxylysine or N^5^-acyl-N^5^-Hydroxyornithine reported by Winkelmann [38]. All hydroxamate siderophores are characterized by peptide linkage [24], except fusarinine C (FsC), synthesized by *Aspergillus nidulans*, which shows ester bonds. Two O_2_ molecules of these groups bind with Fe, known as bi-dentate ligand. Hydroxamate siderophores are capable of binding hexadentate octahedral complex with Fe (III) [39].

Fungi synthesize more than one type of siderophore belonging to a single structural family or different structural families. For instance, *Trichoderma pseudokoningii* and *T. longibrachiatum* synthesize all three structural families of siderophores [40]. The FSR (fusarinine) is a siderophore synthesized by young cultures of *Fusarium roseum*, whereas at the older stage of culture, FSR is replaced by malonichrome. Siderophore identity is a valuable trait in fungal taxonomy [41]. In fact, wide ranges of siderophores are found within a fungal genus. Mor et al. [42] found that siderophore production in *Trichophyton* and *Microsporum* species are similar. For instance, *T. rubrum* and *T*. *mentagrophytes* produced siderophores that are also synthesized by *Microsporum* taxa. The same siderophores, ferrichrome C and ferricrocin, are produced in *Microsporum* species, including *M. canis* and *M. gypseum.* In contrast, *T*. *mentagrophytes* and *T. Tonsurans* produce only ferrichrome. Other fungal siderophores are described in Table 2.

## 3. Biosynthesis of Siderophores and Regulation

Biosynthesis of siderophores is divided into two major pathways: nonribosomal peptide synthetase (NRPS)-dependent, and NRPS-independent [60,61]. NRPSs are large multimodular enzyme complexes. They consist of adenylation domain (A), thiolation domain (T), condensation domain (C), and thioesterase domain (TE). Each module of the NRPS enzymes is responsible for adding amino acids (AAs) and forming peptide bonds. NRPS enzymes determine the sequence and number of AAs in the peptide chain [62]. NRPS recognizes and activates AAs by activating A-domain and acylating adenylate via an ATP-dependent reaction. The next steps are thiolation of (T) domain, in which the activated ester is covalently linked, followed by condensation of the (C) domain, and direct transfer of another acyl amino acid to form a peptide bond [63]. The thioesterase domain (TE) is present in the final unit. The last step consists of assembling or releasing chains from the NRPS by hydrolysis or cyclization. Cleavage of the acyl thioester, which binds to the T domain, is an NADH-dependent reaction [64]. Fungi commonly use four main mechanisms for iron uptake across the cytoplasmic membrane as follows: (i) A shuttle mechanism in which the siderophore bound iron can be taken into the cell and the iron is released by a reductase or by direct ligand exchange in which the recipient siderophore becomes the storage molecule. The gathering ligand is released to capture another iron molecule. This type of transfer has been used by coprogen and ferrichrome families. (ii) A direct transfer mechanism in which iron is taken up (iron is first reduced by the reductive pathway before taken up) while the ligand remains outside the cell. The iron transfer is not a membrane-reductive event but is a membrane-mediated exchange between the gathering siderophore and an internal chelating agent. The transfer mechanism may be by ligand exchange (nonenzymatic) to an internal pool of the chelating agent, which then serves as the storage compound. This type of transfer has been used by rhodotorulic acid. (iii) An esterase-reductase mechanism in which iron ligand is taken up and ester bonds of the iron ligand are split to excrete fusarinine moieties, followed by reduction and storage of ferric iron. This type of transfer has been used by ferric triacetylfusarinine C. (iv) A reductive mechanism with the transport of some ferrichromes, which do not to enter cells but give up ferric iron by reduction with transport of the ferrous iron [36].

**Figure 3:**
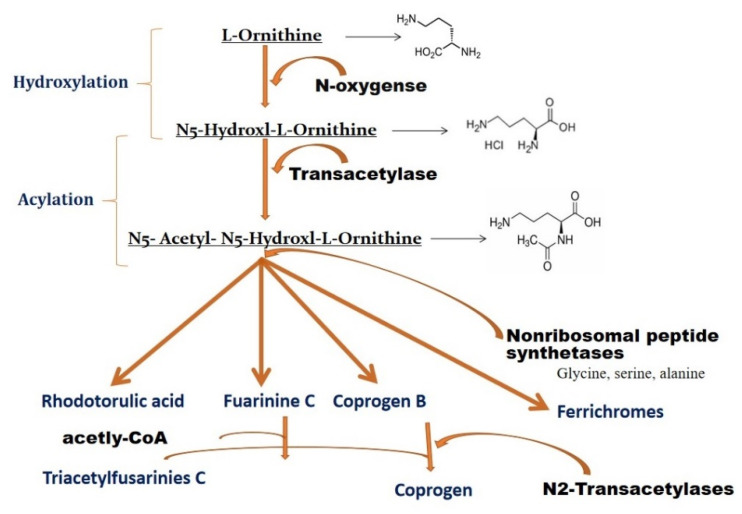
L-ornithine is the biosynthetic precursor of siderophores in fungi. Intermediate steps including hydroxylation, acylation and acetylation lead to the synthesis of Rhodotorulic acid, Fusarinine C and Coprogen (modified and adapted from Philpott [29]).

Hydroxamate fungal siderophores have similar biosynthetic pathways in terms of their basic unit, characterized by hydroxyornithine [65,66]. Figure 3 represents a general schematic biosynthetic pathway [67]. The first step is the hydroxylation of L-ornithine. This is a precursor of siderophores, which is converted into *N*-hydroxy-l-ornithine in a reaction catalyzed by the enzymes L-ornithine and L-ornithine N^5^-oxygenase [61,68]. The second step is the acylation of N-hydroxy L-ornithine to form *N*-acyl-*N*-hydroxy-L-ornithine in the presence of the enzyme transacetylase [67]. The acyl-CoA derivative is an acyl donor, and the reaction is catalyzed by acyl-CoA: N-hydroxy-L-ornithine N-acyl transferase. This step has been reported in *Ustilago sphaerogena* [69,70], while N-acetyltransferase activity in siderophore biosynthetic pathways has been reported in *Rhodotorula pilimanae* [71]. N-acetyltransferase has also been reported in other fungi such as *Fusarium cubense* [72], *Rhodotorula glutinis* [73], and *Aspergillus quadricinctus* [67]. The following steps are the condensation of several N-acyl-N-hydroxy-L-ornithines combined in two to three units and the formation of dipeptides and triesters such as FsC, rhodotorulic acid, and coprogen B [67]. Condensation of amino acid and formation of cyclic peptide FRC were reported in *Aspergillus quadricinctus* [74]. Peptide biosynthesis in siderophore production and respective genes (sid2) have been described in *Ustilago maydis* and *Trichoderma virens* (Psy1) [75,76]. Disruption of these genes makes *U. maydis* and *T. virens* unable to synthesize ferrichrome [76,77].

**Figure 4:**
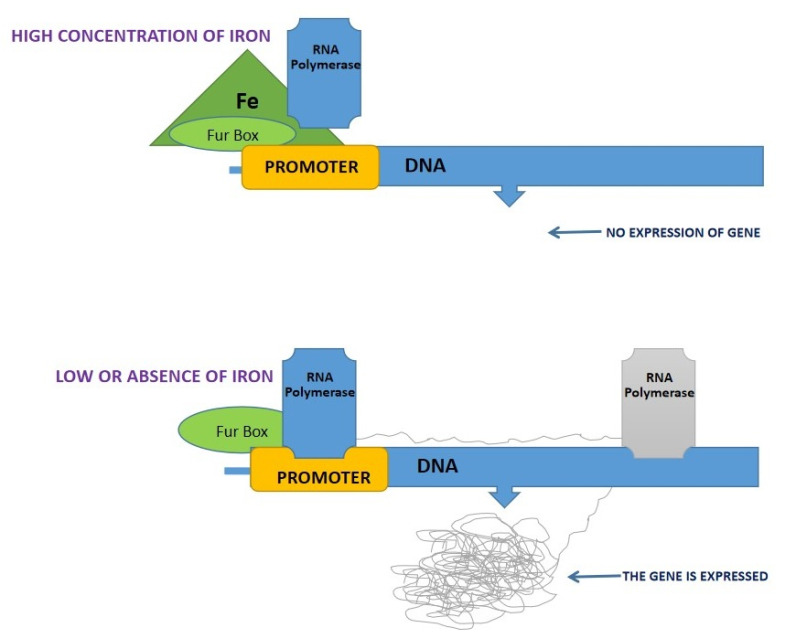
Regulatory model of biosynthesis of siderophore and Fur Box activation or repression: At the high iron concentration, formation of Fur box protein and iron complex in the promoter region of the DNA and RNA polymerases are unable to move forward due to the regression of the gene, while at low concentration of iron, Fur proteins release RNA polymerases leading to the expression of genes.

Transport of iron via siderophores is an investment of biosynthetic energy. The function of siderophores is mainly iron sequestration from an external medium. During excretion, siderophores are released, and a few of them are loaded with iron molecules for supporting the growth of the producing microorganism [78]. Therefore, the excretion of siderophores responds to the availability of iron in the external medium [79]. Fur is a ferric uptake regulatory protein produced in Gram-negative and positive bacteria [80]. A constitutive siderophore mutant in *Salmonella* was observed by Ernst et al. [80], and cloned [81]. The Fur^+^ gene is repressed along with a number of genes involved in iron uptake [82]. The proposed repression model requires the Fur-protein’s internal binding of ferrous iron and then binds to the Fur Box present on the target DNA. Inhibition of RNA polymerase is responsible for the search of the promoter region of the iron-regulatory region. Under limited iron conditions, transport systems and siderophore biosynthesis are activated, and Fur protein is separated from the Fur box on the DNA [83]. In fungi, transcriptional repressor (Fur) proteins are known as GATA factor proteins [84,85]. These proteins contain GATA-type zinc fingers that bind to the siderophore biosynthesis genes [86,87] (Figure 4). Table 3 represents negative fungal regulatory proteins in the biosynthesis and transport of siderophores.

In iron-deficient conditions, *C. albicans* and *S. cerevisiae* use the Aft1 transcription factor to bind with the promoter region of siderophore biosynthetic genes and activate the expression of genes [94,95]. Therefore, external ion concentration is the regulatory factor for intracellular biosynthesis of siderophores and transport proteins in microorganisms. The regulation of siderophore production and transporter proteins are the most important ecological aspects of siderophores. Iron sensing is a crucial step for the survival of competing microorganisms in natural environments. Iron sensing assists the microbes in adapting to ever-changing iron metabolism in different habitats [96]. Therefore, those microorganisms highly sensitive to iron regulation are more resistant to environmental changes. Previous research has provided insight into the low concentrations of iron in environments. Usually, iron-deficient environments are colonized by aerobic microorganism through up-regulation of siderophore biosynthetic genes and transport proteins [97]. The important question here is: where is the low iron content in natural habitats? The marine region, especially in the open oceans, calcareous soils, and freshwater lakes contain iron concentration in surface water in the nano-molar 0,2 to 1 nM range [98] and inhibits growth of plankton, bacteria, and plants. 

In the human body, free iron is absent, and if any iron is present, it is in protein-bound forms such as transferrin or lactoferrin and ferritin [99]. Pathogens can multiply in low iron environments by sequestering iron from host proteins using ferric-binding protein and transferrin-binding protein, as found in *Neisseria*. These transport proteins move from the periplasm to the cytosol [100]. *Pseudomonas aeruginosa* cell lysis and degradation of proteins by the proteases and subsequent iron scavenging by the excretion of siderophores is another method of iron acquisition. There are different routes by which pathogens can utilize iron from the host cells, but the most suitable system is siderophore mediated transport [101]. Another essential aspect of siderophore ecology is the energy saving. The siderophore biosynthesis requires energy, which is obtained from ATP and carbon sources. Siderophore production starts after the germination of conidiospores in fungi [102]. The conidiospores contain a certain amount of siderophores packed into the wall of the spore and secreted at the time of germination [103]. Siderophore genes are responsible for the sporulation in some fungal strains. Knock out of siderophore genes in fungi was shown to inhibit the sporulation process [104]. A notable example is the *Aspergillus fumigatus* conidial siderophore ‘hydroxyferricrocin’, which also aids in germination and oxidative stress tolerance [23]. 

## 4. Siderophore Mediated Iron Transport in Fungi

The fungal cell wall is made up of glucans, chitin, chitosan and glycosylated proteins, and shows a highly dynamic structure [105]. The outer layers of the fungal cell wall are composed of mannoproteins [106]. Mannoproteins affect cell permeability and are influenced by growth conditions. Indeed, mannoproteins allow the passage of nutrients across the cell wall to the periplasmic space and plasma membrane [54]. Regulation and uptake of iron molecules are essential in fungi for maintaining homeostatic processes. As a result, fungi commonly use four main mechanisms for iron uptake, including ferric iron (Fe^3+^) uptake through the production of siderophores, iron assimilation through a redox reaction, heme uptake, and direct iron uptake [8,35]. Every fungal species exhibits an extracellular iron-uptake mechanism known as siderophore-iron transporter (SIT). This SIT, in principle, is constituted by a major protein family that facilitates iron uptake in fungi, acting through the help of the plasma membrane, with high solubility and energy, as a proton-coupled symporter, and releasing iron-chelated siderophore during cell growth. The triacetyl fusarine C (TAFC) and fusarinine C (FsC) have been found to enhance the iron release through partial hydrolysis by the esterase (Estb) enzyme [15].

The structural configuration and properties of siderophores have revealed their vast affinity for iron. All siderophores differ from each other; nonetheless, they share a common conserved structure with a similar functional unit and show an identical pattern of binding to other molecules, i.e., transferrin and lactoferrin. Siderophores typically consist of a peptide backbone that interacts with receptors present in the outer membrane of the cell surface [107]. The hydroxamate siderophores are more structurally complex and hydrophilic. However, denticity plays a more critical role in their affinity toward iron. Most siderophores exhibit a hexadentate structure that allows six coordination sites for ferric ions [10]. An example of hexadentate siderophore is dihydroxylbenzoylserine trimer. It is commonly produced from enterobactin and exhibits preorganized metal-binding via macro cyclization [108].

It has been revealed that the hexadentate siderophores usually have a higher affinity for Fe (III) than tetradentate siderophores. Each of their molecules contains three bidentate ligands fused to form a hexadentate complex. This characteristic also reduces entropic changes during the chelation of a single ferric ion, as compared to the bidentate siderophores, which have only two to three ligand molecules [8,10]. The denticity of siderophores also differs depending on their architecture, and varies from linear dimer to trimer, or cyclic trimer. The cyclization of siderophores, related to the TAFC and enterobactin groups, enhances stability and helps them to resist enzyme degradation [38]. 

Siderophore structure also varies based on the presence of different functional groups. According to Renshaw et al. [109], fungal siderophores are usually formed by the basic unit N*δ*-acyl-N*δ*-hydroxyornithine, i.e., L-isomer of N5-hydroxy-N5-acetylornithine. These siderophores are derivatives of L-ornithine, except for *Neurospora crassa* siderophore. *Neurospora crassa*, obtains a siderophore from neurosporin [51]. All siderophores produced by fungi belong to the hydroxamate group, except the polycarboxylate rhizoferrin. It was revealed by Huschka et al. [110] that the geometrical stability of any siderophore complex depends mainly on the kind and number of its N-acyl residues surrounding the iron coordination center. Well configured stable L-cis ferrichrome siderophores have been identified in *Aspergillus quandricinctus*, *Neurospora crassa*, and *Penicillium parvum*.

The mechanism of siderophore transport is specific or well defined in fungi, which may use multiple transport systems or produce more than one siderophore at a time for efficient recruitment and transportation of metal ions. For example, Howard [15] reported that *Agaricus bisporus* has various transport systems for FSR and FRC, while *N. crassa* has different recognition sites for CPG and the ferrichrome-type siderophore system. *Saccharomyces cerevisiae* retains significant amounts of Fe-chelating molecules, termed as siderophores, in its cell wall and periplasmic space. Under Fe-deficient condition, there is the expression of very high levels of three mannoproteins (FIT1p, FIT2p, and FIT3p) commonly regarded as facilitators of Fe-transport [111]. Siderophore-Fe chelators improve protein retention in the cell wall, whereas deletion of related genes controlling the mannoproteins fit1, fit2, and fit3 reduces ferrichrome and ferrioxamine uptake by 50%. Fe-transport of *S. cerevisiae* is expressed into two genetically separate systems known as reductive and nonreductive systems [29]. The two-step reductive system operates at the plasma membrane level, where the reduction of Fe III (oxidized) into Fe II (reduced) is done by a ferrous-specific complex also known as the high-affinity transporter [112,113].

### 4.1. Ferric Reductase Enzymes (FRE)

**Figure 5:**
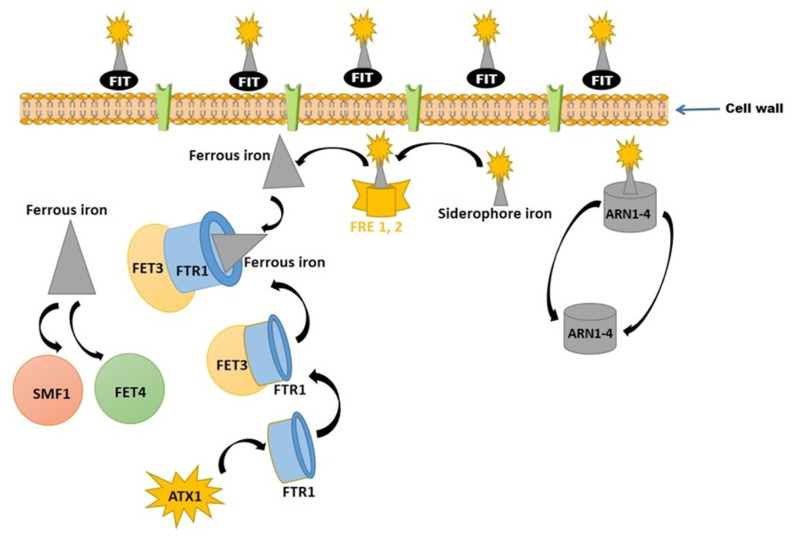
Mechanisms of ferric ion (Fe III) transport in the yeast cell, and subsequent transformation into ferrous ion (Fe II).

The ferric reductase enzymes (FREs) are metalloreductases encoded by FRE1 and FRE2 genes expressed on the plasma membrane, reducing or oxidizing iron and copper [114,115]. These reductases are integral membrane proteins (multiple) and possess binding sites for Fe, coenzymes (FAD, NADPH), and cytochrome (b-types) [116]. *Saccharomyces cerevisiae* exhibited reductases in different forms such as FRE1, 2, 3, 4, 5, and 6 under iron-deficient conditions, and expressed FRE1 and 7, under copper depleted conditions. Fre3p was expressed on the plasma membrane and showed siderophore reductase activity, while Fre6p was located on a vacuolar membrane and involved in reductive export of iron into the cytosol. Initially, ferric citrate reductases (Fre1 and Fre2p) of yeast are characterized by broad substrate specificity and Fe uptake from many ferric sources. Siderophores bind ferric iron with high affinity and convert ferric ion into a ferrous ion, allowing transport by a specific ferrous ion transporter (Figure 5).

The plasma membrane of *S. cerevisiae* is bounded by a porous cell wall, providing the shape of the cells, protecting them from osmotic lysis, and preventing the entry of too large macromolecules. The siderophore-iron retention proteins (FIT mannoproteins) of the cell wall facilitate iron retention with siderophores and do not require siderophore uptake. In a few cases, siderophores cross the cell wall via nonspecific pores. FRE reductases (FRE1,2) reduce Fe (III) into Fe (II) before uptake, and reduced iron is taken up through high-affinity ferrous iron transporters (Fet3p and Ftr1p complex) or low-affinity iron transporters (Fet4 and Smf1). However, the Fet3p requires copper for optimum activity [29]. 

Fre1p and Fre2p catalyze the reduction of several iron-siderophore chelates, including ferrichrome and ferrioxamine B [117]. Fre3p encodes a plasma membrane reductase that catalyzes the reductive uptake of iron bound with hydroxamate siderophores, and Fre4p specifically catalyzes the reductive uptake of di-hydroxamate rhodotorulic acid-iron [118]. The standard reduction potentials of physiological reductants such as NADPH are lower than those of ferric siderophore complexes, which are kinetically unfavorable on the cell surface. However, by coupling reduction of ferric-siderophore with competitive ligand exchange with a ferrous iron-specific chelator at lower pH in hydrophobic environments, the reduction potential can be changed into the level of physiologic reductants [10,119]. Therefore, in vivo ferric siderophores are reduced into ferrous ions at reduced pH in a lipid rich environment. Similarly, transferrin, a high-affinity iron-binding glycoprotein, reduces ferric ion into ferrous ion and removes the reduced form of iron.

### 4.2. Multicopper Permease

Multicopper permease (Ftr1p) is a high-affinity transport complex responsible for transporting reduced forms of iron [120]. The apparent Km of the high affinity enzyme complex is 0.2μM [121] and allows transport at low iron concentration. The ferrous iron is oxidized by Fet3p and requires molecular oxygen [122,123]. The ferric iron is transported into the cytosol via the Ftr1p permease. The oxidase reaction is copper-dependent, and four copper ions are inserted into Fet3p during post-translation in the secretory pathway on the post-Golgi compartment. The copper chaperone Atx1p is responsible for binding and transporting copper to Ccc2p [124]. The copper transporter pumps copper into the post-Golgi vesicle lumen [125]. Both proteins are necessary to maintain adequate cellular copper levels and functioning secretory pathways [126]. These copper-binding proteins, Atx1p and Ccc2p, are synthesized during iron deprivation and not copper deficiency, indicating Fet3-dependent iron uptake. Assembled complexes (Fet3p and Ftr1p) of proteins are retained by quality control systems in the endoplasmic reticulum, if expressed in the absence of their protein partner [127]. The process of ubiquitin-mediated endocytosis rapidly degrades the Fet3p/Ftr1p complex in the presence of high levels of iron [127].

### 4.3. Siderophore-Iron Transporters

Most fungi synthesize and secrete siderophores and small organic compounds that specifically bind with iron molecules with high affinity [7]. Fungal siderophores bind iron with dissociation constants (10^−29^ M), showing greater affinity than any iron-binding ligand in the biological systems. Hsiang [128] reported that all fungi express a nonreductive uptake system specific to siderophore iron chelates. Fungi express transporters with specificity for siderophores secreted by other species of fungi. When the siderophore is abundant, the reductive system of transport can catalyze the uptake of siderophore-bound iron. More than 50% of genes are transcriptionally activated under iron-deprived conditions and are involved in the uptake of iron chelators. The evolution of these uptake systems helps fungi and other microorganisms to compete during the limited availability of iron.

## 5. Biotechnological Applications of Siderophores

### 5.1. Treatment of Infectious Diseases and Anticancer Activity

Siderophores can be used for the treatment of thalassemia [129,130], a disease associated with inherent blood disorder due to abnormal hemoglobin formation. Many studies have shown that compounds such as desferrioxamine B (DFO) are used to reduce this disorder [131,132,133,134]. Rhodotorulic acid, a fungal siderophore, has been investigated as an alternative to DFO for iron and aluminum overload [135]. The mechanism of rhodotorulic acid is similar to the iron excretion of DFO, but increases zinc excretion, resulting in toxicity at the administration site [136]. DFO and other hydroxamate siderophores have been used to treat cancer, malaria actinide contamination, and other infectious diseases [137,138]. DFO was also reported for treatment of acute lymphoblastic leukemia by Estrov et al. [139]. Vergne et al. [140] reported that several other siderophores exhibit anticancerous and antitumor activity. For example, triornicin fungal siderophore, produced by *Epicoccum purpurascens* has an inhibitory effect on tumors in mice [46]. For treating infections, siderophore transport/uptake research can enhance movement of the drugs into the microbial cell conjugated with the siderophore-drug [140,141]. Actinides are radioactive elements known as potent carcinogens. Siderophores and their analogs may enhance the excretion and removal of actinides [142]. However, siderophore research in the medical field is still in progress. Most studies have been concentrated on siderophores of bacterial origin, mainly DFO, and on the siderophore analogs hydroxypyridinones.

### 5.2. Application of Siderophore as Drug Delivery Agents

**Figure 6:**
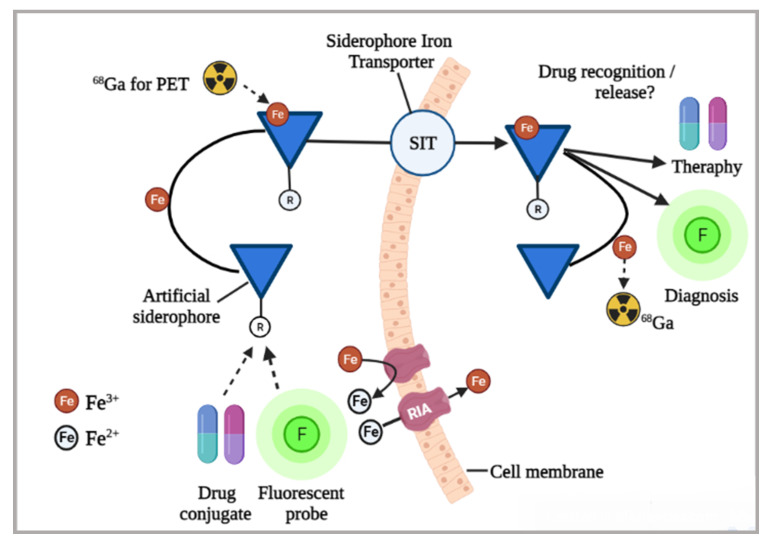
Siderophores for medical applications. The use of different siderophores (natural, artificial, and modified) linked to drugs or fluorescent probes, and ^68^Ga radiolabeled for molecular imaging and/or curative practices. Siderophores facilitated intracellular absorption potentially allows diagnosis through the use of fluorescent or radioactive signal transduction or therapy by introducing antifungal drugs, thus obeying a trojan horse strategy. The siderophores’ metabolic path and translation target is shown by solid line arrows. Substitution of Fe with ^68^Ga (for PET) or drug molecules–or fluorescent probes–siderophores conjugate is indicated by dotted line arrows (modified and adapted from Petrik et al. [149].

Siderophore combined with antibiotic are used as a ‘Trojan horse’ for targeted drug delivery (Figure 6). Using siderophore receptors, this method enables antibiotic transfer across the membrane. *Escherichia coli* was treated with a solution of two arthrobactin-carbacephem conjugates that were created [143]. A siderophore cephalosporin conjugate was examined against several pathogenic bacteria such as *Pseudomonas aeruginosa* and *E. Coli* [144]. In addition, simultaneous treatment with conjugates containing hydroxamic and catechol resulted in bacterial growth inhibition. The sideromycins, which are connected to lorabid or ciprofloxacin, are also of interest [145]. Sideromycins-lorabid conjugate invades the periplasm, whereas sideromycins-ciprofloxacin attacks the cell wall. *Staphylococcus aureus* growth was effectively inhibited by sideromycins. Pyochelin-norfloxacin was another siderophore conjugate compound investigated [146]. Siderophore analogues were developed and connected to an antibiotic-norfloxacin. Among four conjugates, two were found effective against *P. aeruginosa*. A vanchrobactin-norfloxacin conjugate, which demonstrated antibacterial properties against *V. anguillarum* and its variants, is another example of siderophore analogs [147]. Some siderophore-antibiotic conjugates, however, have been shown to promote bacterial growth. For example, *Mycobacterium smegmatis* was not affected by spermexatol-carbacephalosporin conjugates [148].

### 5.3. Application of Siderophores in Vaccine Development

A vibriobactin analogue either connected to bovine serum albumin (BSA) or ovalbumin (OVA) was reported to stimulate the production of antibodies in a mouse model [150]. Based on a murine model of an uropathogenic *E. coli* (UPEC), conjugates of siderophores and antigens were recently exploited for the development of vaccines against urinary tract infections (UTIs) [151]. A decrease in bacterial concentration when mice were given siderophore-cBSA (aerobactin-cationized bovine serum albumin) conjugates, indicated protection via adaptive immunity [152]. Other studies have shown the importance of Fe transport receptors in pathogenic bacteria as vaccine components [153]. An assessment of *E. coli* (O157:H7) siderophore receptors, as well as porin proteins, was carried out on cattle. Two vaccine doses were found to reduce the bacterial prevalence in cattle [154]. As a potent component of vaccine, FhuD receptor of *S. aureus* was examined in another study. Because of the absence of conformational alteration, FhuD-ferrichrome was exploited as the model vaccine antigen [155].

### 5.4. Application of Siderophores in Diagnostics

#### 5.4.1. Radiolabeled Siderophores for Imaging Fungal Infections

Present diagnostic methods such as computed tomography (CT) have severe limitations regarding specificity and sensitivity. For instance, any radiological indication can be associated with fungal infection in CT; thus, novel and better diagnostic methods are required for invasive fungal infections [156]. Siderophore iron transporters (SITs) are potential targets of molecular imaging methods due to their higher upregulation during fungal infection, substrate specificity, and radiolabeled substrate accumulation in the target cell after their energy-driven uptake (Figure 6). In addition, lower molecular mass and high hydrophilicity of siderophores lead to better circulation in infected tissues, speedy clearance from nonspecific tissues and elimination through renal excretion. Another vital characteristic of SITs is the possibility of substituting Fe in the siderophores with an Fe-mimicking radionuclide. Gallium-68 is a diamagnetic isosteric substitution for Fe^3+^ that has been used to describe siderophore complexes extensively [149].

Using only microgram of the siderophore, radiolabeling of a variety of desferri-siderophores with gallium-68 can be achieved, as shown by proof-of-concept experiments [157,158]. *Aspergillus fumigatus* absorption of the siderophore was elevated under iron-deficient conditions and could be inhibited when there was an abundant siderophore or sodium azide, showing that the uptake is selective and energy-dependent. Using PET (Positron emission tomography)/CT technology, pulmonary infection caused by *A. fumigatus* was imaged in a rat model, which revealed a significant buildup of [^68^Ga] Ga-triacetyl fusarinine C (TAFC) in the fungal-infested areas. In sterile inflammations and tumour cells, a considerable accumulation of [^68^Ga] Ga-TAFC was not observed [159]. It was also shown that the use of siderophores is specific to species to a certain level.

Some in vitro research has shown significant uptake of [^68^Ga] Ga-TAFC by *Fusarium solani*, *Rhizopus oryzae*, and *A. fumigatus*, but negligible uptake by *A. flavus*, *Candida albicans*, *A. terreus*, or the bacteria *Klebsiella pneumoniae*, *P. aeruginosa*, and *S. aureus*. In contrast, *A. fumigatus* showed the highest uptake of [^68^Ga] Ga-desferrioxamine-E (DFO-E), followed by *A. terreus*, *F. solani*, *R. oryzae*, *A. flavus*, and the bacterial species *S. aureus* [159]. Altogether, TAFC seems to be fungal-specific in comparison to DFO-E. Therefore, ^68^Ga-labeled siderophores, particularly [^68^Ga] Ga-TAFC, have great potential for imaging invasive *A. fumigatus* infections in patients [149].

Chemical modification of siderophores has been designed for new promising applications. For example, a hybrid imaging compound may be created by binding fluorescent dyes, allowing PET/CT with gallium-68 and optical imaging [149]. To study TAFC identification in *A. fumigatus* by the MirB transporter, initial efforts were undertaken to chemically alter it with positive, negative or neutral charged functional groups [160]. Even without inhibiting fungal uptake, chemical alterations were possible, with encouraging results coming from the diacetylated version of fusarinine C (DAFC), in which functional groups were used to change the free amine. In order to explore the hybrid imaging concept, fluorescent dyes were combined to DAFC based on these results [161]. Using the optical signals, these fluorescent siderophores enable image-guided approaches, such as bronchoscopy or surgical probing. Furthermore, such compounds can be applied for fluorescent microscopy. Specifically, a FsC-Cy5.5 conjugate was used to image the skin infections resulting from *Trichophyton rubrum* [162].

#### 5.4.2. Diagnostics of Siderophore from Urine

The rapid clearance of [^68^Ga] Ga-TAFC through the renal system limits the use of PET to detect infections in the kidney or bladder. These results, instead, sparked research into the use of TAFC as a urine biomarker to diagnose invasive *A. fumigatus* infections. Clinical trials [163] and clinical samples [164] both yielded promising findings. However, additional research is required to validate this strategy prior to its application in the clinic. Moreover, the mass spectrometry approach applied is not often practiced in diagnostic labs. Nonetheless, the presence of TAFC in aspergillosis patients demonstrates that the siderophore system is activated in human infections [149].

### 5.5. Bioremediation of Metal Polluted Environments

Metals have played a vital role in the development of human civilization. However, manufacturing, sludge application, nuclear power plants and mining have caused a serious increase of heavy metal pollution in the environment [165]. In particular, soil heavy metal pollution has become one of the environmental problems of global concern. Siderophores have a strong solubilizing effect on a variety of metals such as Cr, Cu, Ni, Pb, Zn and the actinides Th^4+^, U^4+^ and Pu^4+^ [166]. For this reason, siderophores can contribute to the bioremediation of heavy metal contaminated soil. Although siderophores are mainly used to chelate Fe^3+^, they can be used in the detoxification process of heavy metal pollutants by combining a variety of toxic metals. The ability of siderophores to chelate heavy metals mainly depends on the stability constant of the siderophore and the metal to form a complex [167]. The use of siderophores for remediation of heavy metal pollution has the advantages of low cost, high efficiency, and no environmentally hazardous collateral. In recent years, there has been an increasing interest in the application of siderophores in metal bioremediation. Phytoremediation is an emerging and practical technology in the field of bioremediation, but heavy metal stress can interfere with the absorption and utilization of iron by plants, cause iron deficiency, affect chlorophyll synthesis, turn young leaves yellow, and hinder plant growth. Siderophore-producing rhizosphere fungi are a group of plant rhizosphere growth-promoting microorganisms whose siderophore products can react with iron-containing minerals in the soil to generate soluble Fe^3+^-siderophore chelates, which promote the dissolution of Fe^3+^ in the soil [168]. These rhizosphere fungi can provide nutrients (especially iron) to plants under heavy metal stress, by inducing selective absorption by plants of the different metals available in the environment. In fact, siderophores combined with iron can effectively be absorbed by plant cells, whereas they cannot easily enter the cells when combined with other heavy metals. Therefore, siderophores can significantly alleviate the stress caused by metal toxicity and promote plant growth [169]. In addition, studies have shown that siderophore-producing rhizosphere fungi can maintain their growth-promoting effects on plants in heavy metal polluted soil because siderophores can alleviate the inhibitory effect of heavy metals on the synthesis of plant growth hormones, such as indoleacetic acid, by fungi [170]. Siderophores produced by rhizosphere fungi can chelate with Fe^3+^ to inhibit the absorption of iron by plant pathogens, thus reducing the activity of pathogens, protecting plants from diseases and promoting plant growth [171]. In addition to their growth-promoting effect to improve the biomass of plants, fungal siderophores can also improve the activity of heavy metals in the rhizosphere environment and promote the absorption and accumulation of heavy metals in plants. The activity of heavy metals in the plant rhizosphere is an important factor that determines whether large amounts of heavy metals can be absorbed by plants [172]. The siderophores produced by rhizosphere fungi can combine with heavy metal ions in the soil to form a soluble heavy metal-siderophore chelate, thereby improving the activity of heavy metals in the rhizosphere environment, increasing the absorption and accumulation of heavy metals by plants, and improving the efficiency of phytoremediation [173]. Dahlheimer and colleagues used siderophores to react with oxides containing the heavy metal ions Pt^4+^ and Pd^2+^ and found that siderophores can form soluble chelates with Pt^4+^ and Pd^2+^, thus increasing the solubility of Pt and Pd [174]. Hong et al. reported the in vitro dissolution of copper and zinc via siderophores produced by the fungal species *Fusarium solani* [175]. Other studies have also confirmed that siderophores can promote the dissolution of many common heavy metals, and even metalloids, from their minerals. For instance, siderophores can promote the dissolution of manganese-containing minerals such as Mn_3_O_4_ [176]. At the same time, heavy metals can influence siderophore-producing microorganisms, and the total amount of siderophores increased in copper-contaminated sites [177]. Siderophores and heavy metals can also be stored in fungi after chelation. For example, in soil contaminated by metals, *Hypocrea lixii* can secrete siderophores to accumulate copper and zinc in the biomass [78].

The motor manufacturing industry, sewage sludge, vehicle emissions, and mining are common contributing sources for metal pollution [178,179]. Neptunium and plutonium are man-made actinides present in the environment as pollutants due to the testing of nuclear power stations and weapons production, posing a significant environmental hazard [180]. Siderophores are effective for the solubilization of actinides [181] and form stable tetravalent actinides. They also play a significant role in the mobilization of other metals, including zinc, copper, lead, and cadmium [182,183]. Siderophore-producing microorganisms are abundant in soil [184] and affect the bioavailability of metals and radionuclides present in the environment [183,185]. Siderophores could be used to develop metal recovery or remediation of waste sites, including radioactive waste, due to their complexing ability [167].

### 5.6. Plant Growth Enhancement and Biocontrol of Plant Pathogens

Microbial siderophores provide Fe-nutrition when the bioavailability of iron is limited to enhance plant growth [186], although the mechanism of siderophore mediated Fe-nutrition is still not fully known. However, two possible mechanisms by which plants could obtain Fe from microbial siderophores have been suggested: (i) high redox potential of microbial siderophores can be reduced by the donation of ferrous in the transport system, and (ii) microbial ferric ions are transported in plant root through the apoplast where the reduction of siderophore takes place [187], with consequent ferrous accumulation in the apoplast leading to a high concentration of Fe (II) in the root [188], and (iii) siderophores of microbial origin can chelate Fe from soils and perform ligand exchange with phytosiderophores [189]. These mechanisms depend on several parameters such as concentrations of phytosiderophores, microbial source, pH, and redox potential of root environment [186]. Schenk et al. [190] found that siderophores are a valid eco-friendly alternative to hazardous pesticides. Mycorrhizal fungi are used as biofertilizers for the enhancement of plant growth and development. Higher levels of Fe-sequestration occurred in plants associated with mycorrhizal fungi compared to nonmycorrhizal plants, suggesting that enhanced plant nutrition by mycorrhizal fungi depends on fungal siderophores [191,192]. Plant growth-promoting activities of fungi were previously investigated by Yadav et al. [193] who found that fungal species such as *Trichoderma harzianum*, *Penicillium citrinum*, and *Aspergillus niger* produced siderophores and increased root and shoot length of chickpeas. Siderophores play a significant role in biological control as competitors for Fe to reduce the Fe availability for the pathogens of plants [194]. Wilt diseases of potatoes caused by *Fusarium oxysporum* can be controlled by Pyoverdine siderophores produced by *Pseudomonas* sp. [195]. Apart from fungi, bacterial strains, mainly belonging to the genus *Pseudomonas*, have been extensively studied to improve plant growth by synthesizing siderophores or protecting the plant host from pathogens [196]. 

### 5.7. Enzyme-Inhibiting Activity 

Siderophores are iron chelators capable of inhibiting the iron-dependent activity of enzymes by withdrawing iron. Several studies have shown that ribonucleotide reductase activity is reduced by synthetic siderophores [197] as a result of inhibition of biosynthesis of DNA. In proliferating neoplastic cells, iron delivering transferrin receptors were found to frequently occur on the cell surface and enhance iron requirement by the cell. Inhibition of iron supply by the siderophores reduced the growth of neoplastic cells [132,197]. Therefore, siderophores can be used as inhibitors of cell proliferation and help to design drugs with anticancer activity.

### 5.8. Computational Approaches for the Application of Siderophores

With the advancement in bioinformatics and computational approaches, it has become easier to explore gene clusters for siderophore biosynthetic pathways and their interactions with other proteins and peptides. Siderophores are complex structures of nonproteinogenic amino acids with huge structural variations. These structural variations make siderophores suitable for various applications. Computational and bioinformatics tools help to predict the affinity and properties of siderophores. Norine is a bioinformatics platform that is an easily available unique resource devoted to elucidating the structures of nonribosomal peptides. This tool helps to identify newly discovered siderophores, whether new nonribosomal peptides or variants of an existing family. Similarly, AntiSMASH (antibiotics & Secondary Metabolite Analysis Shell) is an internet-based bioinformatics tool which finds the region in plants, fungi, and bacteria responsible for the biosynthesis of secondary metabolites. AntiSMASH allocates a functional “siderophore” label for biological gene clusters which contain the lucA/lucC gene family specific to the siderophores biosynthetic pathways, which further helps to predict the siderophores activity [198]. These tools help to derive siderophore biosynthesis pathways and make them accessible for reference. The prediction of biosynthetic pathways for the production of siderophores facilitated the discovery of novel and exclusive siderophores such as thioquinolobactin [199]. Molecular dockings and dynamic simulations are additional techniques for analyzing the interaction of siderophores with other proteins. Samsonov et al. [200] successfully applied molecular docking and molecular dynamic simulations to analyze the potential binding interaction between nine bacterial siderophores and lipocaline, a member of human serum a1-acid glycoprotein. A comparative study of computational and experimental results indicated that serum a1-acid glycoprotein can effectively bind with the Fe-BisHaCam and petrobactin, which shows that serum a1-acid glycoprotein can be putatively involved in the nullification of bacterial infections by capturing iron-chelating compounds. Furthermore, Xie et al. [201] explored bioinformatics approaches such as mutasynthesis, genome mining, and activity screening to synthesize fluorinated amychelin fluoroamychelin I siderophores. The resulting fluorinated fluoroamychelin I was able to rescue *Caenorhabditis elegans* from *Pseudomonas aeruginosa*-mediated killing with greater efficiency than traditional antibiotics, including meropenem and ceftazidime. The study showed a successful implementation of bioinformatics approaches for the production of synthetic antibacterial compounds by modifying siderophores. In addition to these, Flux balance analysis (FBA) is a computational tool that is frequently used in metabolic engineering for the improvement of production yield. Siderophores are small-sized metal chelators that are usually secreted in very small quantities by their native microbial hosts. FBA can be applied to predict media composition to enhance production yield that can be further verified experimentally. Recently FBA has been applied to improve the heterologous expression of siderophores by *E. coli* K-12 MG1655 [202]. In conclusion, computational and bioinformatics approaches are helpful to identify novel siderophores, their biosynthetic pathways, and biological activities. Furthermore, these techniques are applicable to modify siderophore structures in order to improve their bioactivity and even their production yield.

## 6. Importance of Siderophores in Nature

### 6.1. Weathering of Soil Minerals

Soil microbes produce siderophores that can stimulate the dissolution of insoluble phases minerals [203,204]. Several mechanisms for siderophore-stimulated Fe dissolution have been proposed [205]. In general, the Fe (III) and siderophore complex forms on the mineral surface and is subsequently transported into the adjacent soil solution, where it is accessible for absorption by microbes and plants [203,206]. Because siderophores and Fe form more permanent complexes, their influence on soil mineral weathering may be greater than low molecular mass organic acids (LMMOAs). Siderophores and Fe (III) form 1:1 complexes with K constants ranging from of 1030 to 1052 [207], whereas, with Fe (III), oxalic acids have constants of K = 10^8^ and citric acids have constants of K = 10^12^ [208]. Nevertheless, when both siderophores and LMMOAs are present, the mineral dissolution rate is increased more than when the solitary siderophore is present [209]. Many studies have reported microbial siderophore involvement in dissolution of Fe minerals due to their relevance in weathering and soil formation. In a study, researchers revealed the effectiveness of hydroxamate siderophores formed by *Suillus granulatus* in dissolving goethite. Due to the continual synthesis of siderophores by *S. granulatus*, significant amounts of Fe (10^9^ mol m^2^/h) were mobilized [210]. Additionally, Sokolova et al. [211] found that fungal siderophores belonging to the family of ferrichrome, including ferricrocin and ferrichrome, contributed to altering the surface structure of biotite and promoting its dissolution in forest podzolic soil.

Compared to a synthetic siderophore, the presence of siderophore-producing actinobacteria, such as *Arthrobacter* and *Streptomyces*, significantly enhanced the Fe dissolution from hornblende [206]. Phytosiderophores have been shown to elevate Fe-containing mineral dissolution, thus contributing to weathering processes of minerals, for example goethite and ferrihydrate [212]. According to Reichard et al. [213], the highest goethite dissolution rate of 1.7 nmol m^2^/h at pH 6 was obtained in the presence of 2′-deoxymugineic acid (phytosiderophores). The transporter genes expression of Fe–phytosiderophore in barley has recently been shown to improve its capacity to remove Fe from soil minerals [214].

### 6.2. Oceanic Biogeochemical Cycle of Fe

Trace metal biogeochemical cycling in the oceans has become a significant source of concern. Fe has gained the most attention out of all the trace metals found in marine waters since it is a critical micronutrient for marine life and affects phytoplankton production and community structure [215]. Marine bacteria are responsible for most organic Fe chelators found in seawater and hence play a central part in the Fe biogeochemical cycle in the ocean [216]. These bacteria compete for Fe with phytoplankton by creating several forms of siderophores, which significantly influence the abundance and solubility of Fe in the ocean [217]. In marine siderophores, either citrate or b-hydroxyaspartate contributes hydroxyl–carboxylate functional groups [198,218]. Siderophores contribute to the photochemical cycle of Fe in surface water by producing complexes of Fe (III)–siderophore that improve the availability of Fe for the phytoplankton [219,220]. Ferrioxamine G was discovered to be broadly dispersed in surface waters across the Atlantic Ocean, while ferrioxamine E was shown to have a more diverse distribution at different depths. These data imply that marine siderophores play a significant part in the biogeochemical cycle of Fe by elevating the abundance, as well as the availability, of Fe in the Atlantic Ocean [221,222].

## 7. Conclusions

Iron is an essential element for all living organisms. Fungi acquire iron from extracellular environments by secreting siderophores, which are low molecular weight, iron-binding molecules. Siderophores play a significant role in the iron homeostasis of fungi, which are similar to bacteria and plants for the mobilization of extracellular iron. Considerable progress has been made on siderophore uptake research and understanding iron assimilation mechanisms in *S. cerevisiae*. However, much more remains to be explored regarding biosynthetic pathways, iron assimilation, and regulation. In this regard, further studies based on genome sequence analysis of fungi and siderophore-mediated iron acquisition in a wide range of fungal species are still needed. Apart from fungal siderophore-type descriptions, several unexplored aspects need to be elucidated, including extracellular excretion mechanisms, details of the siderophore biosynthetic pathways, intracellular iron release, iron metabolism and storage of iron. Iron requirements of fungi open up new research areas for the development of novel antifungal treatments such as iron chelation therapy. Functional studies of siderophores may reveal novel non-ribosomal peptide synthetases that can open the way for the development of new compounds with pharmaceutical value. In fungal species, siderophore-mediated iron uptake is essential for survival as free-living organisms and for establishment of commensal and pathogenic relationships.

Studies conducted on low Fe bioavailability and siderophore activity in different environments may enhance our understanding of siderophore ecology and functions. Metagenomic analyses provide an excellent platform to clarify the structural diversity of siderophores among different fungal species. The knowledge of siderophore biosynthesis and utilization mechanisms in each fungal species could be essential for eradicating pathogenic fungi hiding and replicating in host macrophages.

With the advent of genome sequencing technologies and concurrent omics analysis, there has been a vast increase in our knowledge of siderophore biosynthesis over the past two decades. The first step was identifying gene clusters acting as sources of siderophores via bioinformatics. Prediction of substrate specificity combined with algorithms parsing metabolomic data to link the clusters to corresponding compounds may constitute the following steps. For each step, multiple new techniques have been developed in the last few years. In silico genome mining is an efficient high-throughput approach to uncover potential nonribosomal peptide synthetases (NRPS) genes. Analytical pipelines linking genomics with other omics data have been developed and can reveal much information on the synthesis of such natural products. Computational tools coupled with genome mining provide efficient methods to identify and characterize biosynthetic gene clusters BGCs [223]. Natural product research and siderophore research have been concentrated on bacterial species, and there is an obvious bias in data availability and algorithm development for fungal research. Therefore, it is essential to consider the differences and test the relevance of already developed tools on fungal data before blind usage. It would be ideal to generate, collect, and analyze fungal NRPS data, and particularly reorganize siderophore-producing data for fungal siderophore identification. The lack of such curated data is currently a shortcoming in developing and training prediction/classification models for fungal siderophores. Algorithms for the identification of siderophore-producing BGCs integrated with high-throughput proteomic and metabolomic product detection techniques can lead to the discovery and characterization of novel siderophores.

With the help of computational approaches, inhibitors can be designed against siderophore biosynthetic pathway enzymes and siderophore transporter proteins by applying the principles of structure-based drug design and/or fragment-based drug design. Similarly, the structure-based pharmacophores for these catalytic enzymes can be predicted and searched against the chemical databases for compounds suitable as siderophore biosynthetic enzyme inhibitors. In contrast, biosynthetic pathway enzyme competitive inhibitors can be identified using the shape and structural information of enzyme substrates. Applying shape-based and fingerprint-based similarity searches allows suitable competitive enzyme inhibitors to be predicted from chemical databases. In addition, a drug repurposing approach can be applied to find existing drugs with antifungal activity. This approach reduces drug discovery cost to a great extent, with a higher success rate than other approaches.

## Figures and Tables

**Table 1:** Types of siderophores and their characteristics.

Siderophores	Types	Characteristics	References
Hydroxamates	Rhodotorulic acid	The diketopiperazine ring of N5-acetyl-N5-hydroxy-l-ornithine units linked head-to-head. Produced mainly by basidiomycetous yeasts such as *Rhodotorula* spp.	Haas [35]; Das et al. [36]
	Coprogens	The diketopiperazine ring (dimerum acid) of diketopiperazine ring (dimerum acid) units linked head-to-head. Produced generally by a number of plant pathogens, such as *H. capsulatum*, *B. dermatitidis*, *Fusarium dimerum* and *Curvularia lunata*. These are di or tri-hydroxamates derivatives of rhodotorulic acid with a linear structure composed of trans-fusarinine units.	Haas [35]; Das et al. [36]
	Ferrichromes	Cyclic hexapeptides consisting of tripeptide of *N*-acyl-*N*-hydroxyornithine and three amino acids, serine, glycine and alanine. Several different acyl groups have been found in this family such as acetyl, malonyl, transb-ethylglutaconyl, trans-anhydromevalonyl, and cis-anhydromevalonyl. Ferrichromes are produced by phytopathogenic fungi and by *Microsporum* sp., *Trichophyton* sp., and *Aspergillus* spp. Another function of ferrichromes is the intracellular storage of iron.	Haas [35]; Das et al. [36]
	Fusarinines	Linear or cyclic hydroxamates composed of *N*-hydroxyornithine, which is N-acylated by anhydromevalonic acid. Produced by *Fusarium* spp., *Paecilomyces* spp., and *Aspergillus* spp.	Das et al. [36]
Polycarboxylates	Rhizoferrin	A citric acid-containing polycarboxylate called rhizoferrin has been isolated from *Rhizopus microsporus* var. *rhizopodiformis*. The molecule is composed of two citric acid units linked to diaminobutane. Produced mainly by Mucoromycota, Mucorales (Mucoraceae, Thamnididiaceae, and Choanephoraceae) and Mortierellales (Mortierellaceae), and Entomophthoromycota, Entomophthorales.	Das et al. [35]

**Table 2:** Some examples of fungal siderophores.

Fungal Source	Siderophores	References
*Aspergillus* sp., *Penicillium oxalicum*, *Aureobasidium pullulans*, *Phanerochaete chrysosporium*	Hydroxamates	Ghosh et al. [22]
*Alternaria longipes*	Trihydroxamate (Hydroxycoprogen I, Hydroxyneocoprogen I)	Jalal and Helm [43]
*A. longipes*	Trihydroxamate (N^b^-dimethyl coprogen, N^b^-dimethyl neocoprogen I and N^b^-dimethyl isoneocoprogen	Jalal et al. [44]
*Candida* sp.	Ferrichrome, hydroxamates	Baakza et al. [37]
*Curvularia lunata*	Trihydroxamate (Neocoprogen II)	Hossain et al. [45]
*Epicoccum purpurascens* (Syn. *E. nigrum*)	Trihydroxamate (Isoneocoprogen I or Triornicin)	Frederick et al. [46]
*E. nigrum and C. lunata*	Trihydroxamate (Isotriornicin or Neocoprogen I)	Frederick et al. [46]; Chowdappa et al. [47]
*Fusarium dimerum*	Dihydroxamate (Dimerum acid)	Diekmann [48]
*F. roseum*	Cis-fusarinine	Emery [49]
*dimerum*	Trans-Fusarinine	Diekmann [48]
*F. dimerum*	Trihydroxamate (Coprogen B)	Diekmann [48]
*Gliocladium virens*	Fusarinine A, Fusarinine B	Jalal et al. [50]
*Neurospora crassa*	Neurosporin	Eng-Wilmot et al. [51]
*Penicillium* sp.	Trihydroxamate (Coprogen)	Pidacks et al. [52]
*Penicillium* sp.	N, N’N’-triacetylfusarinine C	Moore and Emery [53]
*Paracoccidioides* sp.	Hydroxamates	Lesuisse et al. [54]
*Rhizopus microsporus*	Carboxylates (rhizoferrin)	Drechsel et al. [31]
*Rhodothamus chamaecistus*	Fusarinine C (FsC), Fusigen	Haselwandter et al. [55]
*Saccharomyces cerevisiae*	Catecholate, hydroxamate, ferrioxamine, ferricrocin	Senthilnithy [56]
*Trichoderma* sp.	Trihydroxamate (Pamitoylcorprogen)	Anke et al. [40]
*Trichoderma* sp.	Hydroxamates, carboxylates	Baila et al. [57]
*Ustilago inflorescentiae*	Dihydroxamate (Rhodotorulic acid)	Atkin and Neilands [58]; Muller et al. [59]

**Table 3:** Negative fungal regulatory proteins in the biosynthesis and transport of siderophores.

S.No	Regulatory Protein Similar to GATA Factor	Organisms	References
1.	URBS1	*Ustilago maydis*	Voisard et al. [88]; An et al. [86]
2.	SRE	*Neurospora crassa*	Zhou et al. [89]
3.	SREP	*Penicillium chrysogenum*	Haas et al. [90]
4.	SREA	*Aspergillus nidulans*	Haas et al. [91]; Oberegger et al. [24]
5.	GAF2p	*Schizosaccharomyces pombe*	Hoe et al. [92]; Pelletier et al. [93]

## Data Availability

Not applicable.

## Abbreviations

TAFC	triacetyl fusarine C
FsC	fusarine C
FSR	fusarinine
CPG	coprogen
Frr	ferricrocin
FRC	ferrichromes
Fsg	fusigen
NRPS	nonribosomal peptide synthetases
SIT	siderophore-iron transporter
Estb	esterase
FIT	facilitator of iron transport
FREs	ferric reductase enzymes
Fet	ferrous iron transporter
DFO	desferrioxamine B
